# Walking capacity and its association with quality of life among children with down syndrome in Saudi Arabia

**DOI:** 10.1186/s12887-023-04519-8

**Published:** 2024-01-19

**Authors:** Saad A. Alhammad, Amani S. Alqahtani, Khalid S. Alwadeai, Maha F. Algabbani, Adel A. Alhusaini

**Affiliations:** Department of Health Rehabilitation Sciences, College of Applied Medical Sciences, King Saud University, P.O. Box 10219, Riyadh, 11433 Saudi Arabia

**Keywords:** Down syndrome, Walking capacity, Children, Quality of life, Physical function, Social function, Emotional function, School function

## Abstract

**Background:**

Walking ability, which has been connected to better health and independence, is one of the daily activities that is negatively impacted by Down syndrome. Thus, the objective of this study was to examine the walking capacity and its association with the quality of life of children who have Down syndrome compared to those who do not have Down syndrome in Saudi Arabia.

**Methods:**

For this cross-sectional study, we recruited 68 Arabic-speaking children aged 6 to 12 using a convenience sampling method from August to November 2021. Children were divided into two groups: those who do not have Down syndrome (*n* = 38) and those who have Down syndrome (*n* = 30). Children in the Riyadh region of Saudi Arabia who do not have Down syndrome were chosen randomly from two schools. Children who have Down syndrome were selected from multiple associations and centers in the same region. A 6-minute walk test was used to measure the child’s walking capacity. The Arabic version of the Pediatric Quality of Life Inventory scale was used to assess the child’s or parent’s perceptions of the child’s quality of life and its physical, emotional, social, and school functioning domains.

**Results:**

The difference in the mean 6-minute walk test scores between children who have and who do not have Down syndrome was statistically significant, with a mean difference = 105.6, 95% confidence limit = 57.2—154.0, *p* < .0001. The linear regression analysis after adjusting for age, height, weight, and body mass index revealed that walking capacity was found to be significantly associated with the worst score on the Pediatric Quality of Life Inventory scale (β = −2.71, SE = 0.49, *p* < .0001) and its domains of physical, social, and school functioning (β = −2.29, SE = 0.54, *p* < .0001; β = −2.40, SE = 0.58; *p* = .001; β = −3.71, SE = 0.56, *p* = .002, respectively) in children who have Down syndrome, but they had better emotional functioning than children who do not have Down syndrome.

**Conclusions:**

Children who have Down syndrome were less able to walk and were highly associated with the worst possible quality of life, which included the lowest levels of physical, social, and school functioning. Early interventions with techniques must be developed to improve the quality of life for these children.

**Supplementary Information:**

The online version contains supplementary material available at 10.1186/s12887-023-04519-8.

## Background

Children who have Down syndrome, also known as trisomy 21, have varying degrees of mental and physical difficulties [1], such as digestive and cardiac problems [2]. As the world’s population rises, the lifetime prevalence of Down syndrome is also rising. For example, the prevalence of Down syndrome in the United States rose from 3.3 per 10,000 people in 1950 to 6.7 per 10,000 people in 2013 [3]. In 2015, it was projected that 4.9 out of 10,000 individuals in Europe and 3.3 out of 10,000 individuals in the former Eastern Bloc countries would have Down syndrome [3]. Saudi Arabia has a lower incidence of Down syndrome (1 per 554 live births) than the rest of the world (1.25–1.67 per 1,000 live births) [4]. However, the number of individuals with Down syndrome in Saudi Arabia has been steadily rising as a result of a decreased death rate [5].

For various reasons, including congenital cardiac problems, muscle hypotonia, poor cardiovascular fitness, decreased muscle strength, poor coordination and balance, and intellectual disability, children who have Down syndrome can have markedly inferior walking capacity [6, 7]. In contrast with typical children, they can train more gradually to learn, acquire motor skills, and enhance their quality of life [8]. Additionally, prior studies have suggested that parental care and support, medical direction, and community-based support systems, such as inclusive education at all levels, may help children who have Down syndrome live their lives to the fullest [9, 10].

Children who have Down syndrome often have delayed walking because they are slower to achieve early motor milestones, such as grabbing, rolling, sitting, and standing [11]. Nonetheless, the capacity to walk has been associated with a child’s level of independence and good health in children who have Down syndrome, indicating that it can be a reliable measure of quality of life [12]. Some recent studies [11, 13, 14] examined the associations among balance, gait, functioning, and quality of life in children who have Down syndrome. However, only one study [15] has examined walking ability in male Saudi Arabian children who have Down syndrome. Findings from that study reported that boys who have Down syndrome in Riyadh, who were 8 to 12 years old, had much less ability to walk than children who do not have Down syndrome. Therefore, this study aimed to examine the walking capacity and its association with the quality of life of children who have Down syndrome compared to those who do not have Down syndrome in Saudi Arabia.

## Materials and methods

### Study design and participants

For this cross-sectional study, we recruited Arabic-speaking children aged 6–12 years using a convenience sampling method from August to November 2021. After sending the participants an invitation letter outlining the purpose of the study, we accepted children with parents or guardians who voluntarily agreed to have them participate. All children were divided into two groups: those who do not have Down syndrome (*n* = 38) and those who have Down syndrome (*n* = 30). The group who did not have Down syndrome consisted of 38 children of the same age with no medical issues or recent injuries recruited from two randomly selected local schools. The other group included 30 children who have Down syndrome, who were recruited from randomly selected various associations and organizations in Riyadh, Saudi Arabia. The numerous organizations included the King Khalid University Hospital, the Saudi Association for Special Education, the Efada Center for Down Syndrome, the National Center for Early Intervention, and support groups for families and children who have Down syndrome.

We selected children for recruitment from Riyadh because it is one of Saudi Arabia’s major cities and has a population that is typical of the country’s cities in terms of socioeconomic diversity. Children who have Down syndrome were excluded if they had undergone any lower limb surgery in the past, used a walking aid, had moderate to severe cardiac problems, or had any other condition or injury that might have affected their physical function (such as cerebral palsy). Children from both groups were excluded from the study if they did not complete the accelerometer-wearing period [16].

### Walking capacity

The 6-Minute Walk Test (6MWT) was utilized to evaluate each child’s walking capacity. It is a practical test that measures a person’s walk distance in six minutes and is used to evaluate functional exercise capacity in several cardiopulmonary conditions, including Down syndrome [17]. This test, which also serves as a predictor of morbidity and death, has been widely employed in rehabilitation programs to assess walking capacity [18]. The 6MWT was carried out over a minimum of a 12-meter-long open area, including corridors and the schoolyard, following defined procedures [19] and wheel-measuring equipment protocols [20]. Before beginning the 6MWT, the participants rested for 10 min. The researcher then took their vital signs to rule out any conditions that may make the 6MWT hazardous, such as a heart rate > 120 beats per minute, blood pressure > 180 mm Hg +/− diastolic 100, or oxygen saturation > 85% [17]. Pulse oximetry was used to measure heart rate and blood oxygen saturation [21]. In pediatrics, blood pressure is measured with a digital arm sphygmomanometer [22].

The children and their parents were instructed to sit on a chair with a back and armrests. The arm used to take the measurement was supported at the sternum. Along the walking course, a measuring tape with a cone at each end (to indicate the turning point) was set up at 1-meter intervals. The participants were instructed to move around these cones as naturally and easily as possible. Throughout the exam, the researcher followed each individual closely and recorded each round that was completed. After the test session, the data were reported to the nearest meter. A stopwatch and trundle wheel were used for this test to measure the distance traveled. The 6MWT is a reliable and valid test with an interclass correlation coefficient of 0.84 for individuals with Down syndrome [17].

### Quality of life

The Pediatric Quality of Life (PedsQL) inventory scale [23], which has an Arabic translation, was used to assess how a child or parent perceived the quality of their child’s life on four general core scales (domains): physical functioning (PF, 8 items), emotional functioning (EF, 5 items), social functioning (SF, 5 items), and school functioning (ScF, 5 items). There are two Arabic versions of the PedsQL for children aged 5–7 [24] and 8 or 12 years old [23]. Each version contained two sections: one to be completed by the child (in a parallel child self-report format) and the other by the child’s parents (in a parent proxy report format). In both groups, each child was represented in this study by their parents.

There were some minor differences between the versions and sections. The version for children aged 5‒7 years has three choices for each item, where the answer represents 4 (“almost always”), 2 (“sometimes”), and 0 (“not at all”). The other versions have five choices for each item (0 = “never a problem,” 1 = “almost never a problem,” 2 = “sometimes a problem,” 3 = “often a problem,” and 4 = “almost always a problem”). In addition, there is a difference in the simplicity of wording to suit the target age group, despite the similarity of meaning between them. The results of both versions are converted to a score of 0–100. The score for each domain is calculated using the following formula: the sum of the items is divided by the number of items answered by the participant. The overall quality of life score is calculated by summing all the items reported in all domains, where 100 represents the best quality of life. The quality of life scores (PedsQL) were categorized into three groups: worst (< 0.50), moderate (0.50‒0.80), and high (> 0.80) [25]. The PedsQL scale is a reliable (good internal consistency of alpha = 0.9) and valid (Spearman’s rho range between 0.27 and 0.70) instrument for assessing the quality of life of children with Down syndrome [26].

### Covariates

The researcher used a screening sheet to gather demographic data. The child’s weight was determined while they were wearing minimal clothing and no shoes. The Eufy Body Sense Smart Scale was used twice to measure the weight to the closest 0.1 kg. With the headset in the Frankfurt plane and heels firmly planted against a wall, height was measured twice and recorded to the nearest 0.1 cm using a wall-mounted stadiometer. Weight in kg divided by height squared (m^2^) is the formula for calculating body mass index (BMI).

### Sample size

Using the G*POWER statistical software (version 3.1.9.4; Universitat Kiel, Germany), the minimum required sample size was determined to be 70 children (35 for each group), with a medium effect size of 0.5 (based on Cohen’s *d*) and a significance level of 0.05. and a power level of 0.80, with the addition of 10% to account for the likelihood of drop-off [27].

### Statistical analysis

The Kolmogorov–Smirnov test was used to determine the normality of the continuous variables [28]. The participants’ sociodemographic and anthropometric characteristics were summarized using means and standard deviations for continuous data, while frequencies and percentages were used for categorical data. Significance differences between groups (those who have and those who do not have Down syndrome) were examined using the chi-squared test for categorical data and the independent t-test, analysis of variance, and post hoc test for continuous data.

The difference in 6MWT scores between children who have and who do not have Down syndrome was assessed using an independent t-test. The Pearson correlation coefficient was used to examine the correlations and collinearity of age, height, weight, and BMI with quality of life, PF, EF, SF, and ScF. The correlation coefficient scores were defined as high (> 0.70), moderate (0.50–0.69), low (0.30–0.49), and negligible (< 0.30) [29].

A linear regression analysis adjusted for age, height, weight, and BMI was used to examine the association between walking capacity and quality of life in children who have Down syndrome compared to those who do not have Down syndrome. All analyses were conducted using the Statistical Package for Social Studies (SPSS) version 19 for Windows (IBM SPSS, Chicago, IL, USA). All results were considered statistically significant at a *p-*value of 0.05.

### Ethics approval

The Declaration of Helsinki’s principles guided the conduct of this study. The King Saud University College of Medicine’s ethical committee approved this study (No. 20/0673). Before collecting data, each eligible child and their parent or legal guardian signed an informed consent form regarding their participation.

## Results

**Fig. 1:**
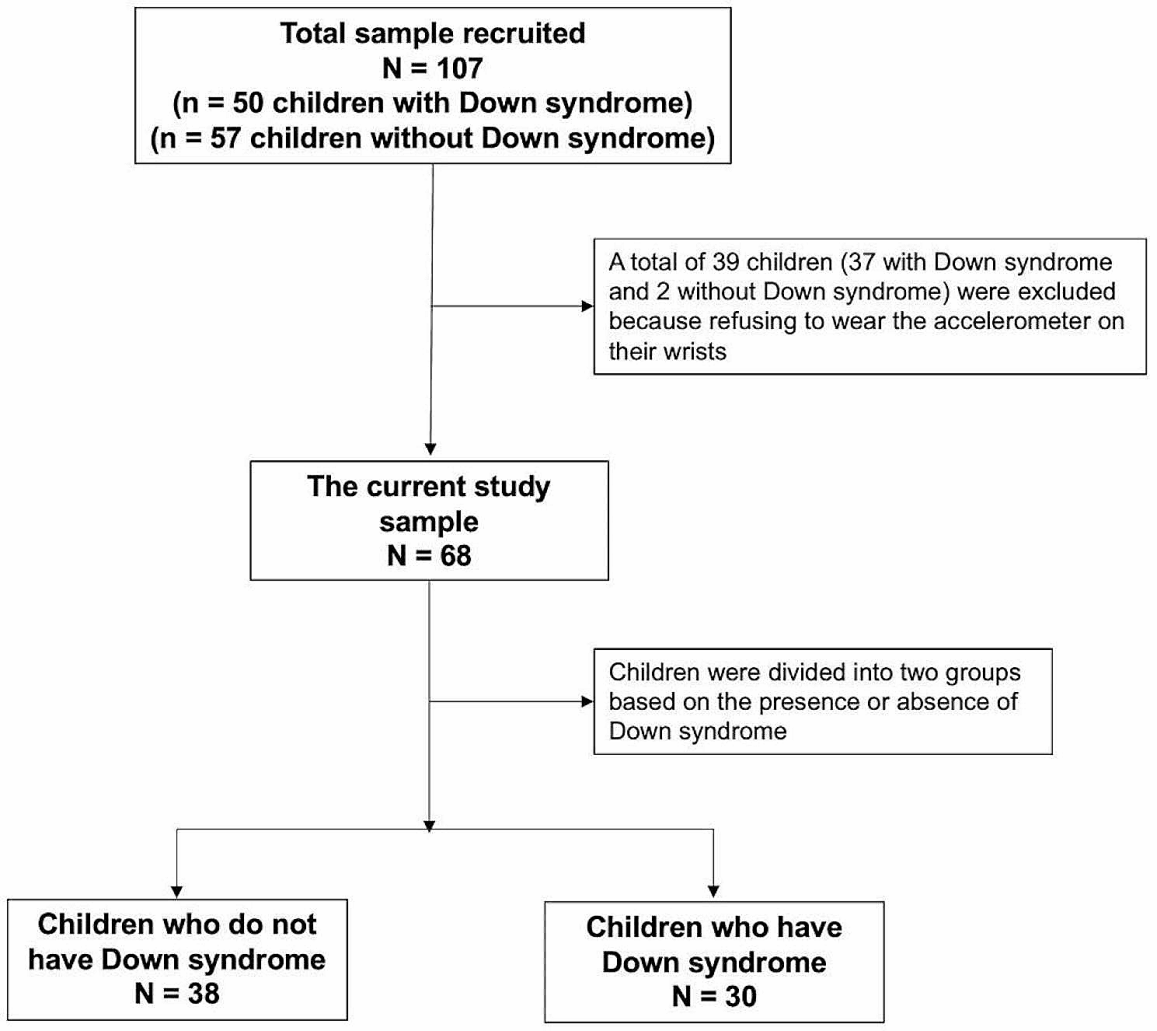
The flow of the study sample

Of 107 children who have and who do not have Down syndrome, 68 (63.6%) were included in this study, after excluding 39 (36.4%) who refused to wear the accelerometer on their wrists. Out of these 68 children, 38 (55.9%) did not have Down syndrome, and 30 (44.1%) had Down syndrome (Fig. 1).

**Fig. 2:**
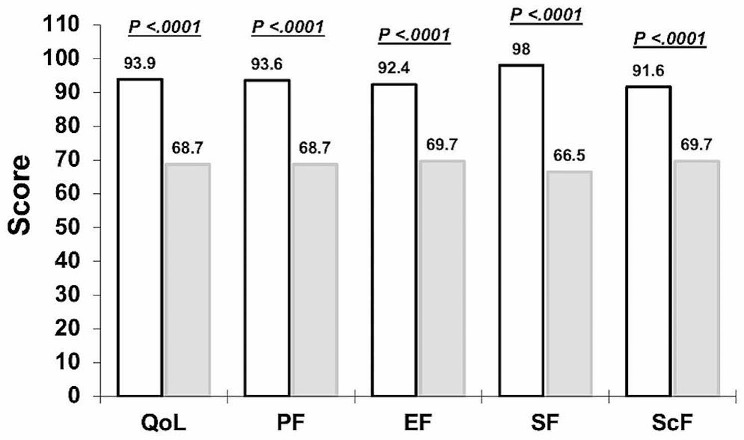
The average quality of life and sub-scale scores in children who do not have Down syndrome (no fill) and who have Down syndrome (filled)

In the study, the average age of the children who have and who do not have Down syndrome was 9.8 years. The height and BMI differed significantly (*p* < .05) between the groups. More children in both groups were significantly right-hand than left-hand dominant (*p* = .043). Most of the parents of children who have Down syndrome had a monthly income of 12,000 or more Saudi riyals (48.6%). Most of the children who have Down syndrome had no education (27.9%) and lived in their own houses (39.7%) (Table 1). Figure 2 shows the average PedsQL and sub-scale scores for the children who have and who do not have Down syndrome.

**Table 1:** Characteristics of the study sample

Characteristics	Total *N* = 68	Groups		p
		Children who do not have Down syndrome *n* = 38 (55.9%)	Children who have Down syndrome *n* = 30 (44.1%)	
Age in year, mean ± SD	9.8 ± 2.1	9.8 ± 1.9	9.8 ± 2.4	*0.973*
**Sex**, n (%)				*0.064*
Boys	38 (55.9)	25 (36.8)	13 (19.1)	
Girls	30 (44.1)	13 (19.1)	17 (25)	
Weight in kg, n (%)	32.7 ± 13.1	33.6 ± 11.8	32.8 ± 14.5	*0.799*
Height in cm, n (%)	129.3 ± 14.5	134.3 ± 14.7	124.3 ± 14.4	*0.006*
BMI in kg/m^2^, n (%)	19.1 ± 4.5	18.1 ± 4.0	20.2 ± 5.0	*0.058*
**Dominant hand, n (%)**				*0.043*
Right	62 (91.2)	37 (54.4)	25 (36.8)	
Left	6 (8.8)	1 (1.5)	5 (7.3)	
**Parent’s income per month in Saudi riyal, n** (%)				*0.027*
<3000	1 (1.5)	1 (1.5)	0 (0.0)	
3,000‒6,000	10 (14.7)	10 (14.7)	0 (0.0)	
6,001‒9,000	12 (17.6)	5 (7.4)	7 (10.3)	
9,001‒12,000	10 (14.7)	4 (5.9)	6 (8.8)	
>12,000	35 (51.5)	18 (26.5)	17 (48.6)	
**Child education, n** (%)				*< 0.0001*
None	19 (27.9)	0 (0.0)	19 (27.9)	
Kindergarten	3 (4.4)	0 (0.0)	3 (4.4)	
Pre-school	38 (55.9)	34 (50)	4 (5.9)	
Elementary school	8 (11.8)	4 (5.9)	4 (5.9)	
**Mother’s level of education, n** (%)				*0.209*
None	5 (7.3)	4 (5.9)	1 (1.5)	
Intermediate school	3 (4.4)	1 (1.5)	2 (2.9)	
High school	16 (23.5)	6 (8.8)	10 (14.7)	
Bachelor	44 (64.7)	27 (39.7)	17 (25)	
**Father’s level of education, n** (%)				*0.292*
None	1 (1.5)	1 (1.5)	0 (0.0)	
Intermediate school	3 (4.4)	1 (1.5)	2 (2.9)	
High school	15 (22.1)	11 (16.2)	4 (5.9)	
Bachelor	49 (72.1)	25 (36.8)	24 (35.3)	
**Home type, n** (%)				*0.752*
Apartment	5 (7.3)	2 (2.9)	3 (4.4)	
Villa	58 (85.3)	33 (48.5)	25 (36.8)	
Floor	5 (7.3)	3 (4.4)	2 (2.9)	
**House type, n** (%)				*0.033*
Own house	53 (77.9)	26 (38.2)	27 (39.7)	
Rental	15 (22.1)	12 (17.6)	3 (4.4)	

The difference in the 6MWT mean scores between children who have and those who do not have Down syndrome was statistically significant, with a mean difference = 105.6, 95% confidence limit = 57.2—154.0, *p* < .0001 (Table 2). Low positive correlations were found between the sub-scale scores and BMI, 6MWT, and PedsQL (Table 3). After adjusting for age, height, weight, and body mass index, walking capacity was significantly associated with a lower quality of life (*β* = −2.71, standard error [SE] = 0.49, *p* < .0001), PF (*β* = −2.29, SE = 0.54, *p* < .0001), SF (*β* = −2.40, SE = 0.58, *p* = .001), and ScF (*β* = −3.71, SE = 0.56, *p* = .002) in children who have Down syndrome but had better EF than those who do not have Down syndrome (Table 4).

**Table 2:** The 6-meter walk test score in children who do not have and who have Down syndrome

Exposure	Groups		MD [95% CL]	t-value	p
	Those who do not have Down syndrome Mean [95% CL]	Those who have Down syndrome Mean [95% CL]			
6-meter walk test	368.3 [332.6, 404.0]	262.7 [230.5, 294.8]	105.6 [57.2, 154.0]	4.35	*< 0.0001*

**Abbreviations**: MD, mean difference; CL, confidence limit

**Table 3:** Correlation coefficients for children who do not have and who have Down syndrome

Variable	QoL		PF		EF		SF		ScF	
	r_s_	p	r_s_	p	r_s_	p	r_s_	p	r_s_	p
Age	0.02	0.860	0.04	0.697	0.05	0.653	0.12	0.315	0.15	0.219
Height	0.27	0.021	0.30	0.012	0.18	0.144	0.22	0.069	0.05	0.674
Weight	0.09	0.43	0.06	0.608	0.29	0.014	0.19	0.107	0.24	0.041
BMI	0.32	0.006	0.32	0.007	0.21	0.078	0.35	0.003	0.25	0.037
6MWT	0.35	0.002	0.34	0.004	0.31	0.010	0.38	0.001	0.63	< 0.0001

**Abbreviations**: *r*_*s*_, Pearson correlation coefficient; 6MWT, 6-meter walk test; PF, physical function; EF, emotional function; SF, social function; ScF, school function; QoL, quality of life

**Table 4:** Regression analysis of the association between walking capacity and quality of life in children who have Down syndrome

Outcome	β	SE	P
***Quality of Life (QoL)***			
Intercept	29.32	73.3	*0.691*
Children who have Down syndrome	−2.71	0.49	*< 0.0001*
*R* ^*2*^	0.48		
***Physical Function (PF)***			
Intercept	46.9	81.0	*0.564*
Children who have Down syndrome	−2.29	0.54	*< 0.0001*
*R* ^*2*^	0.43		
***Emotional Function (EF)***			
Intercept	72.4	87.1	*0.409*
Children who have Down syndrome	−2.40	0.58	*0.001*
*R* ^*2*^	0.35		
***Social Function (SF)***			
Intercept	46.1	84.5	*0.587*
Children who have Down syndrome	−3.71	0.56	*< 0.0001*
*R* ^*2*^	0.52		
***School Function (ScF)***			
Intercept	39.5	100.9	*0.696*
Children who have Down syndrome	−2.69	0.67	*0.002*
*R* ^*2*^	0.28		

**Abbreviations**: *β*, estimate; SE, standard error

## Discussion

This study examined walking capacity and its association with the quality of life of children who have Down syndrome compared to those who do not have Down syndrome in Saudi Arabia. The findings from this study indicate that children who have Down syndrome had a lower walking capacity and were significantly more likely to have the worst quality of life, including PF, SF, and ScF. This is the first study in Saudi Arabia to examine the association between walking ability and quality of life in boys and girls aged 6 to 12 years old who have Down syndrome.

The results of this study are consistent with a previous study [15] conducted in Saudi Arabia, which found that boys 8 to 12 years old who have Down syndrome in Riyadh had significantly lower walking capacity than those who do not have Down syndrome. The fact that the two groups differed significantly in terms of height and BMI could be one explanation. According to a previous study [30], the variation in body size as a child develops is one of the key elements determining walking patterns.

In this study, the children who did not have Down syndrome were taller and had lower BMIs than those who had Down syndrome. This supports a study [31] that found people who had Down syndrome to be noticeably shorter than their typically developing counterparts. According to another investigation [32], more children who had Down syndrome were likely to have high BMI scores—up to 30%—than those who do not have Down syndrome (17%) and those with other intellectual deficits (12–30%). A prior study [33] found a substantial correlation between the 6MWT and several demographic factors, including height and BMI.

The walking ability of children who have Down syndrome in Saudi Arabia is unknown. Only one prior study [15] showed that Saudi children who have Down syndrome, aged 8 to 12 years, had lower walking ability than those with average development. The average 6MWT score in the current study was 262.7 m, less than the 342.1 m reported in the prior study [15]. Moreover, other recent studies [34, 35] that were published outside Saudi Arabia also revealed an average 6MWT of 372 m in Mexico and 571 m in Sweden, which is greater than the children’s walking ability in the current study. Nevertheless, those studies included adults, teenagers, and obese children who have Down syndrome and also had undetected heart conditions and various other conditions. Despite these conditions being highly prevalent in this community, they may have had an impact on the performance and outcomes of the 6MWT.

More research is required in Saudi Arabia to document the link between the children’s ability to walk and their quality of life. For instance, a recent study [36] in Saudi Arabia looked at children with and without congenital heart disease (CHD) who had Down syndrome and were between the ages of 5 and 15 years. The findings revealed that children who have Down syndrome and CHD had a similar quality of life to those who did not have both conditions. Another recent study [37] found that caring for children who have Down syndrome considerably negatively influenced their quality of life in various areas. Additionally, recent studies outside Saudi Arabia [10, 38, 39] emphasized reporting quality of life rather than its association with walking ability.

### Study strengths and limitations

The main advantage of this study is that it is the first of its kind to look at walking ability and its association with the quality of life in Saudi Arabian children aged 6–12 years who have Down syndrome. Another strength was that this study utilized the widely used and valid 6MWT and PedsQL scales to assess the walking capacity and quality of life of the participants, respectively.

There were some limitations to the current investigation. An investigation of causal linkages was not possible because the study was cross-sectional. The limited sample size could hamper the validity of this study. As a result, the results need to be read and applied cautiously. The findings are not necessarily generalizable to the rest of the Saudi population because the study was conducted in the city of Riyadh. One of the study’s other weaknesses was that pain was not measured during walking. Following the test, a few of the children may have complained of minor foot issues and muscle soreness, but this was not recorded for the study. In the future, it would be important to record and examine this aspect of the testing as well. However, it has been found that pain during the 6MWT is far more common in adults, especially in obese individuals compared to lean ones [35]. The 6MWT is impacted by the corridor’s length [19]. The test was administered to the normal-weight children in the current study in a 12-meter corridor. A long corridor produces fewer turns, which could result in a longer 6-meter distance [19]. Children with normal weights should have benefited from this, but even so, the data showed that they traveled a lesser distance.

## Conclusions

The purpose of this study was to examine the walking capacity and its association with the quality of life of children who have Down syndrome compared to those who do not have Down syndrome in Saudi Arabia. The results demonstrated that children who have Down syndrome had a reduced capacity to walk, and it was linked to the worst quality of life in terms of their ability to operate in the social, academic, and physical domains. Rigorous planning, initiative, and early intervention are necessary to improve the quality of life for Saudi Arabian children who have Down syndrome. To completely comprehend the working capacity of these individuals and how it relates to quality of life, as well as to apply the findings to the Saudi Arabian population as a whole, more research with a larger sample size is necessary.

### Electronic supplementary material

Below is the link to the electronic supplementary material.

Supplementary Material 1

## Data Availability

The data that support the findings of this study are available from the corresponding author upon reasonable request.

## Abbreviations

BMI: Body mass index
CHD: Congenital heart disease
EF: Emotional functioning
PF: Physical functioning
PedsQL: Pediatric quality of life
ScF: School functioning
SE: Standard error
SF: Social functioning
SPSS: Statistical package for social studies
6MWT: 6-meter walk test
