# Supplementary material for: Walking capacity and its association with quality of life among children with down syndrome in Saudi Arabia
Source: BMC Pediatr. 2024 Jan 19;24:55. doi: 10.1186/s12887-023-04519-8 (PMC10797893; doi:10.1186/s12887-023-04519-8)
Supplement: Supplementary file 1 — Supplementary Material 1 [file 12887_2023_4519_MOESM1_ESM.pdf]

SCRIBENDI

Certificate of Editing and Proofreading

This certifies that a version of the document titled

Walking capacity and its association with quality of life among children with Down syndrome in Saudi Arabia

authored by

Saad Aziz

was edited and/or proofread by Scribendi as order number

975840

for clarity, consistency, and correctness according to the requirements and guidelines specified by the client.

Mon, 06 Nov 2023

Scribendi Inc

SCRIBENDI INC.
405 RIVERVIEW DRIVE
CHATHAM, ON N7M 0N3 CANADA
+1 (519) 351 1626

Printed on 2023-11-08 at 11:42 AM
